# Farmer preferred traits and genotype choices in *Solanum* a*ethiopicum* L., Shum group

**DOI:** 10.1186/s13002-021-00455-y

**Published:** 2021-04-13

**Authors:** Brenda Nakyewa, Godfrey Sseremba, Nahamya Pamela Kabod, Moses Rwothtimutung, Tadeo Kyebalyenda, Kenneth Waholi, Ruth Buteme, Mildred Julian Nakanwangi, Gerard Bishop, Elizabeth Balyejusa Kizito

**Affiliations:** 1 Department of Agricultural and Biological Sciences, Faculty of Science and Technology, Uganda Christian University Mukono, P.O. Box 4, Mukono, Uganda; 2 National Institute of Agricultural Botany, East Malling Research, Kent, UK

**Keywords:** African indigenous vegetables, Farmer trait preferences, Variety adoption

## Abstract

**Background:**

*Solanum aethiopicum* L. is a nutrient dense African indigenous vegetable. However, advancement of its improved varieties that can increase productivity, household income, and food security has not been prioritized. Further still, studies on some of the crops that have been worked have indicated that it is not a guarantee that the improved varieties will be accepted by the end users and therefore there is need to identify and profile what genotypes are of interest to farmers and their preferred traits through inclusive participatory evaluations.

**Methodology:**

Farmer participatory evaluations were conducted to profile farmers’ traits of interest and preferred genotypes. A total of 24 genotypes were established in three replications in 6 farms in 3 districts; Wakiso, Mukono, and Luwero as these are the major producing districts of the vegetable in Uganda. A total of 177 sex-disaggregated farmers were engaged in scoring the genotypes for pest, disease and drought tolerance, general appeal, leaf yield, leaf texture, and seed yield for best 10 genotypes under each variable.

**Results:**

Non-significant differences in trait (*p* > 0.05) and genotype preferences (*p* > 0.05) were obtained between men and women. The most desired farmer traits were seed and leaf yield, followed by pest and disease resistance. The overall preferred genotype in terms of disease and pest resistance, leaf yield, leaf texture, and seed yield were E12 followed by E11.

**Conclusion:**

Gender does not seem to influence farmer choices for the *S*. *aethiopicum*, Shum group, indicating an opportunity for single variety prototype advancement by breeders and dissemination by seed companies.

**Supplementary Information:**

The online version contains supplementary material available at 10.1186/s13002-021-00455-y.

## Background

African eggplant (*Solanum aethiopicum* L.) [1] is classified into four morphological groups based on use namely Gilo, Shum, Kumba, and Aculeatum [2, 3]. Gilo and Shum are cultivated for their fruits and leaves, respectively. Kumba is cultivated for both fruits and leaves while Aculeatum is ornamental. The Gilo group is globally cultivated while Shum is most common in Uganda [4], Nigeria, and Cameroon. In Uganda, the Shum group is locally known as ‘Nakati’ where it earns livelihood to over 4,000,000 people in urban and peri-urban areas. In this study, we focused on the Shum group (leafy type) which is culturally, nutritionally, and economically integrated with several communities in Uganda; and the crop has in previous studies been referred to as African eggplant Shum, *Solanum aethiopicum* Shum, *S*. *aethiopicum* Shum or simply Shum by Sseremba et al. [5–8] who suggested this naming based on works of Adeniji et al. [2, 3]. To emphasize, the Shum group being a leafy vegetable, is cultivated for its leaves which are used as a necessary accompaniment to staple foods in Africa contributing a rarely appreciated food and nutrition security especially for vulnerable populations [9, 10]. *S. aethiopicum* is much appreciated bitter flavor which is preferred for sauce to go with banana cake (matoke) in Buganda. Culturally, it is associated with widely held beliefs in marriage ceremonies.

*S. aethiopicum* being an AIV has received less attention in research and productivity is low. Among the causes of low productivity include drought susceptibility [5, 6], pests and diseases, limited access and availability of quality seed, and high post-harvest deterioration [11, 12]. Development and advancement of improved varieties can increase productivity, household income, and food security [13]. For example, improved potato varieties in Nepal yielded more than the local varieties (15.4 ton/ha and 13.1 ton//ha) [14, 15]. However, it is not always obvious that farmers will adopt the improved varieties; therefore, it is important to engage them as end users during the variety development and evaluation process [16].

A number of factors may account for a low adoption of new varieties; this is because the interests of the breeders or researchers may not match famers’ preferences since they are always multivariate in nature [17]. For example, when improved potato varieties that are tolerant to diseases and highly yielding were developed in Ethiopia, the adoption rate was only 23% and this was probably because yield was not the only trait of preference by farmers [18]. While there is ready market for *S. aethiopicum*, farmers may not necessarily or rapidly adopt improved varieties unless fronted genotypes meet market’s interests [14, 19]. Further, the major variety selection criteria for vegetable breeders are pest and disease resistance, yield and taste preference; however, these are non-exhaustive and may not be applicable to understand location specific preferences [20]. This scenario creates a challenge to breeders to always work with the end users right from definition of breeding goals through variety development and testing as well as feedback [20, 21].

Participatory variety evaluations (PVE) bring together in a field setting to rank traits of importance such as yield, quality traits, resistance to pest, and disease resistance [20]. PVEs enable breeders have a deeper understanding of traits that are relevant to both the farmers for informing breeding goals [22, 23]. PVEs underpin optimistic changes in farmers’ perceptions and willingness to adopt the new varieties as their subjective preferences for instance in relation to gender for the specific characteristics maybe considered during the breeding process [24]. Considering gender in PVE or gender disaggregated participatory plant breeding is also key in order to take care of the varied preferences [25]. For example, when evaluating millet varieties in Botswana, women’s traits of interest were yield, early maturity and ease of hand harvesting while men only considered yield and quality of the straw [24]. Kolech et al. [20] also noted that, when evaluating potato varieties in Ethiopia, men participants were more concerned with market related traits while women attached importance to suitability of sequential harvesting. Limited information is available for *S. aethiopicum*, Shum group on farmer preferred traits and genotypes. This study therefore focused on identifying preferred leaf morphological traits and profiling *S. aethiopicum*, Shum group genotypes preferred by the farmers. The research questions asked were (i) which *S. aethiopicum*, Shum group genotypes meet farmer expectations and why? (ii) What are the farmers’ *S. aethiopicum*, Shum group preferred traits and why?

## Materials and methods

### Study area and target population

The study was conducted in central Uganda in the districts of Wakiso (longitude 00^o^24′N, Latitude 32^o^32′E), Luwero (longitude 00^o^50′N, Latitude 32^o^30′E), and Mukono (longitude 00^o^20′N, Latitude 32^o^45’E). The three districts were selected because they are the leading *S*. *aethiopicum* producing areas in Uganda [19]. This area is dominated by the Baganda tribe. All the areas receive bimodal rainfall with rain peaks in months of March and November. The study purposively selected small-scale farmers who typically grow *S*. *aethiopicum* as a vegetable or seed or both. *S*. *aethiopicum* in Uganda is typically grown by small scale farmers whose holdings average 2 acres.

### Study design

A mixed methods approach was used to get genotype preferences from the different African eggplant farmers. Quantitative data was collected by farmers physically evaluating plots with different genotypes. Qualitative data was then collected sex disaggregated focus group discussions to get the elaborations for the farmers’ traits of preference [26].

### Sampling method and sample size

Multistage sampling was used in the study. Purposive sampling was used to obtain the leading districts and farmer groups in each district for *S*. *aethiopicum*, Shum group vegetable production. Cluster sampling was then applied whereby commercial farmers (both men and women) were separately selected from each farmer group to participate in the study. A total of 177 farmers participated in the evaluation process. Six key informants (a man and a woman from each district) who were lead commercial farmers were purposively selected and six sex disaggregated focus group discussions were conducted (two from each location).

### Field layout and planting

Six fields per district with three replications were used to grow 24 different *S. aethiopicum* Shum group genotypes. The study genotypes have previously been described for morphological distinctiveness by Sseremba [12]. Each plot per field had four rows with a spacing of 60 cm between rows and 30 cm within rows. Evaluations were made on the two middle rows. Fields in Wakiso and Luwero were planted in August 2019 while that in Mukono was planted in November 2019, due to relative differences in onset of the rains for planting.

### Data collection

#### Genotype selections

At vegetative stage of crop growth between the seventh and eighth week after planting, the farmers were engaged in individual selection by ranking their best ten performing genotypes out of 24. Ranking was based on farmer perceptions of damage by pests and disease, leaf yield, general appeal, drought resilience, and seed yield. Field participatory evaluation sessions were guided by researchers and consisted of 10 farmers per group. Each group evaluated all plots, and thereafter they were disaggregated by gender to form new focus group discussions over their selections.

#### Farmers traits of preference

Sex disaggregated focus group discussions of not more than 10 participants were conducted (2 per location) to get an in-depth understanding of farmer preferences to get farmers traits of interest in *S. aethiopicum.* Farmers mentioned important traits for fresh leaf produce and for seed. Information on how they decide during selection was also picked. Further, two key informant interviews of lead farmers (a man and a woman) per location were also conducted. A lead farmer in this study was one who had grown the vegetable every year for at least 10 years. Farmers mentioned the key traits they use to identify their ideal Nakati vegetable. They also identified common pests and diseases that attack *S. aethiopicum* and how they were controlling them. The traits were then grouped into vegetable production and market preferences. Vegetable production traits included pest and disease resistance, drought tolerance while market preferences included general appeal, leaf yield, and leaf texture.

### Data analysis

#### Farmer genotype preferences

Scores made by the individual farmers on disease resistance, pest resistance, drought tolerance, general appeal, leaf yield, leaf texture, and seed yield were entered in MS-Excel (2016) and exported to GenStat 12th edition for *F* test on farmer preferences at 5% significance level. Mean squares of farmer scores were computed to get relationships and interactions between location, gender, and genotype. Cross tabulations were then used to get variety preferences per location and overall preferences. The overall mean of the six attributes was computed and the overall (average) preference score of a given genotype was estimated. The best ten farmers’ overall genotype preferences were got by getting the mean of the first and second scores for each parameter and then getting the mean of means. Using the overall mean, then 10 best ranked (scored) genotypes were considered.

#### Farmers traits of preference

Qualitative data was recorded verbatim, transcribed, and coded. Themes on traits of preference were used to identify traits preferred by men and those preferred by women. Themes on traits for vegetable production, seed production, market preferences, and common pests and diseases were also made.

## Results

### Participant characteristics

**Fig. 1:**
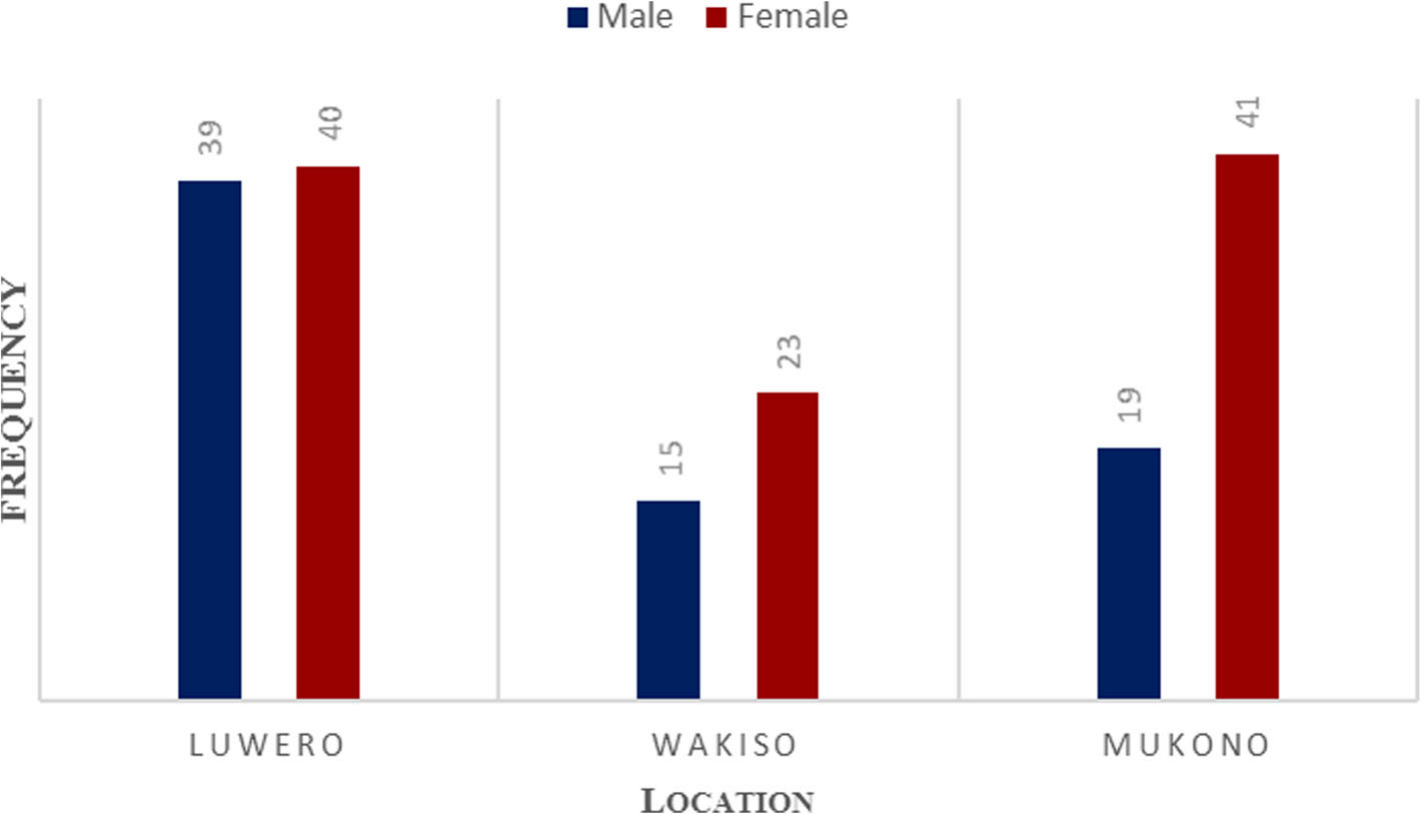
Number of participants per district

Seventy-nine farmers from Luwero district (39 men and 40 women), 38 from Wakiso (15 men and 23 women), and 60 from Mukono (19 men and 41 women) participated in the study (Fig. 1). Participants mentioned that *S. aethiopicum* is a nutritious sauce that is prepared even on ceremonial functions. “Although we have other vegetables, S. aethiopicum is the most fronted side sauce on functions such as graduation parties and wedding ceremonies.” Male key informant—Mukono.

### Farmers’ genotype preferences

#### Location, gender, and genotype effects

There was a very highly significant difference between genotypes (*p* < 0.01) for all parameter preferences, and location by genotype preferences (*p* < 0.001) (Table 1). Choices between men and women, however, were non-significant (*p* > 0.05). As detailed in Table 3, E12 emerged the overall most preferred by participants (30 participants) followed by E11 (24 participants), E15 (19 participants), E9 and E18 (14 participants), E1 (13 participants), E4 and E2 (12 participants), E7H and E14GP (11 participants).

**Table 1:** Mean squares of farmer scores for the different variables considered among genotypes across test locations

Source of variation	d.f	DRS	PRS	PVS	LYS	LPS	SYS
Location (L)	2	2.45	1.10	2.21	0.24	1.11	0.72
Gender (S)	1	2.08	0.19	11.44	8.90	0.16	0.06
Genotype (G)	23	34.97***	26.42***	62.38***	35.50***	27.99***	19.46***
L × S	2	1.43	1.17	2.63	0.68	1.47	0.45
L × G	46	18.48***	16.18***	17.37***	16.55***	14.76***	22.21***
S × G	23	9.61	8.07	9.47	6.27	10.12	4.79
L × S × G	46	7.52	8.62	6.48	8.65	8.91	5.82
Error	2702	7.84	7.76	7.70	7.86	7.92	7.95

*DRS* disease resistance score, *PRS* pest resistance score, *PVS* general appeal score, *LYS* leaf yield score, *LPS* leaf preference score, *SYS* seed yield score

***Significance at 0.01 error margin

#### Location specific preferences

In Luwero District, E12 emerged the most preferred for being disease resistant (Table 2), pest resistant, highest leaf yield, and favorable leaf texture, while E11 was scored as most vigorous and E5 was the highest seed yielding genotype. The overall most highly scored genotype in Luwero District was E12 followed by E11 as shown in Table 2. In Wakiso District, E1 was scored the most disease resistant genotype. E6 was the most pest resistant, E12 was ranked the most vigorous, E12 was the most leaf yielding, favorable leaf texture was E12, and the highest scored genotype for seed yield was E2. The overall most preferred genotype in Wakiso District was E12 followed by E1, E2, and E4. In Mukono District, E12 was the most scored as a disease resistant genotype, E15 was scored the most pest resistant, E11 was the most vigorous, and E12, E11, and E18 were scored the most leaf yielding. For texture preference, E12 was the most scored and E15 was scored the most seed yielding genotype. The overall preferred genotype in Mukono District was E12 followed by 15 and E11.

**Table 2:** Farmers’ genotype preferences based on selected plant attributes at Luwero, Wakiso, and Luwero districts in Uganda

Geno name	Disease	Pest	Vigor	LYS	LPS	SYS	Overall
**Luwero**							
E12	20	14	19	19	13	7	15
E11	14	11	20	17	11	4	13
E15	12	10	12	8	10	5	9
E9	10	7	6	4	10	5	7
E7H	6	8	10	5	7	4	6
E14GP	8	6	4	7	5	4	5
E3S	5	7	9	5	4	4	5
E19	9	6	4	7	5	1	5
E5	1	6	2	4	3	17	5
E18	6	5	7	5	4	3	5
**Wakiso**							
E12	4	3	10	8	9	3	6
E1	13	6	2	2	5	4	5
E2	5	5	4	2	4	9	5
E4	7	3	5	4	3	6	5
E11	5	3	9	4	4	2	4
E6	1	7	7	5	3	3	4
E18	8	2	2	4	2	3	3
E20	0	1	6	6	4	3	3
E3S	2	1	5	5	4	2	3
E14GP	1	3	5	2	4	3	3
**Mukono**							
E12	12	9	11	8	11	3	9
E15	6	10	10	6	6	7	7
E11	7	8	13	8	6	2	7
E18	7	8	5	8	4	5	6
E13	8	5	4	7	6	3	5
E14G	6	5	5	6	4	6	5
E3H	5	7	6	7	4	4	5
E8	6	8	4	5	5	5	5
E9	3	3	5	6	6	7	5
E16	3	6	4	4	7	5	5

*LYS* leaf yield score, *LPS* leaf preference score, *SYS* seed yield score

#### Genotype selections per trait

##### Disease resistance score

For disease resistance, participants scored E12 as the most resistant followed by E11, E1, E18, E15 and E9, E4, E14GP, E7H, and E2 as number 10. Under the second-choice score, the best genotypes were E12, E15, E11, E4, E18 and E1, E2, E9, E14GP, and E7H. Getting the mean of the first and second scores, E12 became the most disease resistant followed by E11, E15, E1, E18, E4, E9, E2, E14GP, and E7H took the tenth position (Table 3).

**Table 3:** Overall performance of all evaluated genotypes based on average number of farmers choosing a genotype as their first and second priority in respect to selected plant attributes

Genotype	Disease			Pest			Vigor			Leaf yield			Leaf texture			Seed yield			Overall
	1	2	Mean	1	2	Mean	1	2	Mean	1	2	Mean	1	2	Mean	1	2	Mean	
E12	44	27	36	30	20	25	58	22	40	49	19	34	35	30	33	14	11	13	30
E11	26	24	25	18	25	22	47	35	41	27	29	28	25	16	21	4	11	8	24
E15	20	26	23	20	25	23	19	24	22	10	21	16	17	20	19	12	13	13	19
E9	20	13	17	16	9	13	14	11	13	13	11	12	20	16	18	7	19	13	14
E18	22	17	20	13	14	14	7	19	13	17	15	16	7	12	10	14	6	10	14
E1	24	17	21	13	11	12	7	6	7	7	17	12	12	10	11	12	21	17	13
E4	16	19	18	9	8	9	13	7	10	11	11	11	8	13	11	10	19	15	12
E2	11	16	14	9	10	10	8	10	9	6	14	10	7	12	10	15	23	19	12
E20	5	10	8	6	8	7	8	13	11	15	13	14	15	8	12	12	19	16	11
E3S	8	11	10	9	8	9	11	22	17	16	11	14	9	12	11	6	11	9	11
E14GP	13	10	12	11	14	13	8	12	10	11	13	12	13	8	11	8	13	11	11
E7H	12	7	10	17	14	16	15	15	15	11	11	11	8	9	9	9	7	8	11
E19	8	18	13	10	11	11	12	6	9	13	11	12	8	8	8	16	10	13	11
E5	6	3	5	12	9	11	5	5	5	6	7	7	9	10	10	32	19	26	10
E10	7	10	9	4	12	8	8	8	8	10	9	10	12	8	10	19	14	17	10
E8	11	8	10	14	11	13	5	9	7	11	13	12	7	9	8	13	13	13	10
E13	10	16	13	7	10	9	5	12	9	10	10	10	8	12	10	8	8	8	10
E14G	8	5	7	9	7	8	5	10	8	13	11	12	5	9	7	12	11	12	9
E3H	9	7	8	10	11	11	11	12	12	11	10	11	12	3	8	5	4	5	9
E6	8	7	8	16	11	14	15	11	13	4	13	9	5	5	5	9	4	7	9
E7S	5	5	5	7	6	7	3	11	7	7	7	7	8	4	6	18	16	17	8
Local check	3	2	3	12	9	11	4	5	5	8	5	7	5	11	8	18	12	15	8
E16	6	7	7	7	14	11	4	6	5	9	8	9	7	11	9	10	7	9	8
E17GP	7	7	7	6	9	8	10	9	10	7	8	8	7	9	8	8	6	7	8

##### Pest resistance score

E12 was ranked number 1 by 30 participants as being the most pest tolerant, followed by E15, E11, E7H, E9, E18 and E1, E14GP, and E4 and E2. Under the second choice, E11 and E15 were second best followed by E12, E18, E7H, E14GP, E1, E2, E9, and E4 was the tenth. Considering the mean for the first and second scores, E12 was ranked the most persistent followed by E15, E11, E7H, E18, E9 and E14GP, E1, E2 and E4 score the tenth position.

##### General appeal (plant vigor score)

Genotypes with the highest scores were E12 (58 participants) followed by E11, E15, E6 and E7H, E9, E4, E19, and E3H and E3S. Under second choice score, the best genotypes were E11, E15, E12, E18, E7H, E14GP, E9, E2, E4, and lastly E1. The mean score of the two scores ranked E11 as the best vigorous followed by E12, E15, E7H, E9 and E18, E4 and E14GP and E1 as the tenth preferred genotype.

##### Leaf yield score

E12 received the highest score as the best choice for the highest leaf yield (58 participants) followed by E11, E18, E9, E4, E7H, E14GP (11 participants), E15, E1, and E2. For the second choice, E11 received the highest score (29 participants) followed by E15, E12, E1, E18, E2, E14GP, and E9, E4, E7H. Taking mean scores, E12 emerged most selected (34 participants) followed by E11, E15and E18, E9, E1, E14GP, E4 and E7H, and E2.

##### Leaf texture preference scores

Genotypes that received the highest number of participants that scored them as best were E12 (35 participants) followed by E11, E9, E15, E14GP, E1, E7H and E4, E18 and E2. Under the second-choice score, the best scored genotypes were E12 (30 participants), E15, E11 and E9, E4, E18 and E2, E1, E7H) and E14GP. The mean score of the two scores ranked E12 as the most preferred followed by E11, E15, E9, E14GP, E4, E1 (11 participants), E18 and E2, and E7H was scored tenth.

##### Seed yield scores

The E2 genotype received the highest number of participants that scored it as the best seed yielding variety (15 participants). This was followed by E12 and E18, E15 and E1, E4, E7H, E14GP, E9, E11). For genotypes that were ranked as the second-choice score, E2 received the highest number of participants (23 participants), followed by E1, E9 and E4, E14GP and E15, E11 and E12, E7H, and E18. Getting the mean of the first and second scores, E2 received the highest number of participants (19 participants) followed by E2, E4, E9, E15, E12 (13 participants), E14GP, E18, E11, and E7H.

### Ranking of important traits by farmers in *S. aethiopicum* Shum group

**Fig. 2:**
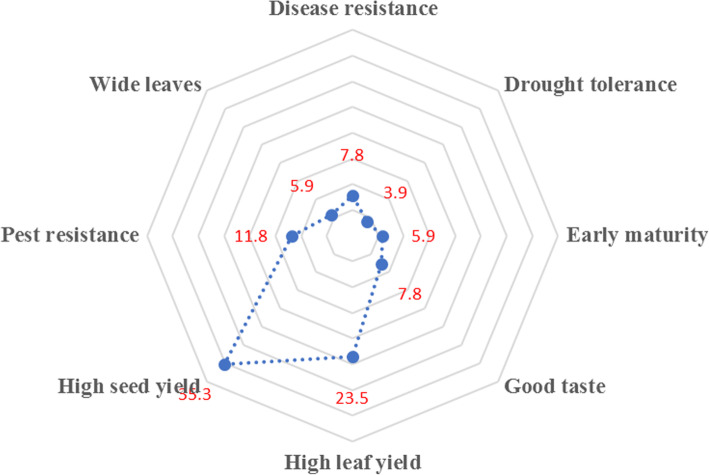
Relative preference of different *S*. *aethiopicum* traits by farmers

During the focus group discussion, a list of leaf morphological traits was mentioned that included leaf size, number of leaves, general appeal, damage by pest and disease, time. Thirty-five per cent of the farmers preferred a genotype with high seed yield in *S. aethiopicum* Shum group production (Fig. 2). These found it more profitable to grow for seed rather than for vegetable leaf in the market. “Although the seed market is still low, I prefer a genotype that gives more seed yield because the profit from seed is more than the vegetable.”—male key informant, Mukono. For this reason, these farmers selected vegetables that were highly branching or that grew tall. The potential for high seed yield perceived as the more the branching, the more fruits borne. “We prefer the branched variety that grows tall because it gives more seed yield”—women FGD Luwero. 23.5% of the farmers grew the vegetables for leaf production and leaf yield was important. They mentioned that highly branching genotypes and plant height were important traits in selecting such vegetable lines. “We prefer a variety that gives so many branches and grows tall because the more the branches, the more the leaves and the more income.”—women FGD, Mukono.

This was followed by pest resistance (11.8%). “When pests attack the plant, they eat leaves and stalks and they do not grow again reducing leaf and seed yield.”—female key informant, Mukono. Furthermore, “Management of the pests is expensive and therefore we get low profits.”—men FGD, Wakiso. Farmers also mentioned that seed which is infested does not germinate. “Seed that affected by fruit rot does not germinate and once a buyer takes, they will never buy from you again.”—female FGD, Wakiso. Furthermore, infested vegetables are also not bought on the market. Common pests mentioned by participants included maggots, grasshoppers, lady bird beetle, cut worm, aphids, monkeys, spider web, caterpillar, and mites.

Participants (7.8%) also liked disease resistant genotypes since diseases affect the quantity and quality of the yield. Common diseases mentioned by farmers that attacked *S. aethiopicum* included fruit rot, wilt, and leaf spot. Participants (7.8%) liked *S. aethiopicum* Shum with a good taste (the one that is not bitter).

Early maturity was also a trait mentioned by participants (5.9%). They preferred a variety that takes a shorter time to mature (6 weeks for the vegetable and 12 weeks for seed). Farmers (3.9%) also liked a genotype that takes a short time (3 to 4 min) to get ready when cooked.

Figure 2 also indicates that drought tolerance was among the traits which farmers wished to have (3.9%). “One of the major constraints to S. aethiopicum production is drought and this has greatly affected yield because the general appeal is poor.”—men FGD, Mukono. To women, irrigation was a burden, “the water sources are far and so irrigation is a very big challenge to us and therefore we shall be glad to get a genotype that is drought resistant.”—women FGD, Wakiso.

## Discussion

### Farmers genotypes of preference

E12 had the highest score for diseases and pest tolerance, vigor, leaf yield, and leaf texture. This genotype showed no sign of disease and pest infestation in all farmer fields. Whereas E12 had the best rank, an earlier sensory taste by consumers gave a feedback that the genotype is bitter (unpublished data). It is perceived to be culturally degrading to beneficiaries and unethical issue by researchers to advance a non-palatable, poor taste, or low-quality food item in society. This consequently leaves us with options to forward E11 and E15 as the farmers’ choices. The criteria on taste- and visual-based choice presents a disconnect between consumer and farmer goals. During key informant interviews, other traits such as cooking time and time to maturity emerged to be of interest by the farmers. Common diseases mentioned by farmers that attacked *S. aethiopicum* were not differing from findings by Dinssa et al. [25] on common pests for *S. aethiopicum.* Participants mentioned that pests affect the yield and quality of both the vegetable and seed.

### Farmer preferred traits

Seed yield came out as the most important trait as it was mentioned by majority of the participants. Participants mentioned that they preferred a genotype that gave high seed yield because growing for seed was more profitable than the vegetable. Preference for seed yield created another need for seed market linkages. Farmers also preferred genotypes that gave many branches and grew tall since many branches yield more fruits and hence much seed. This is corroborated by findings of Jameson and Song [27] who reported that branching had a positive correlation with seed yield. Abady et al. [28] and Banla et al. [29] studies on farmer ground nut trait preferences in Ethiopia and Togo, respectively, also indicated a positive correlation of branching with seed yield.

Farmers also preferred a genotype that yields many and wide leaves as this would be the best for commercial vegetable production. This relates to Adeniji and Aloyce [30] and Diallo et al. [31] findings on farmer preferences for Ethiopian mustard and sorghum in Ethiopia and Mali, respectively. The vegetable market also prefers wide leaves [19] and therefore farmers prefer a variety that will have a ready market.

The disease and pest resistance traits were liked because pests affect the quality of the vegetable leaves, hence attracting low prices. Farmers also incur a lot of expenses managing the pests and diseases cumulating on the cost of production. This trait is similar to the characteristics preferred by Sorghum farmers in Zimbabwe [32].

Farmers’ preference for drought tolerant genotypes was because drought lessens the vigor of the plant affecting its yield [4, 5]. This is in agreement with farmer traits of preference for rice varieties in Kenya [33]. The finding also relates to farmer preferences for ground nut and cowpea varieties in Togo and Namibia [29, 34]. To women, drought is a major challenge because they find it hard to fetch water for irrigation since most of the fields are not near sources of water.

Taste was also a desired trait because the market prefers mild bitter taste (tasty or good taste) genotypes and imperatively farmers like the trait since they produce for the market. Farmers are also the first market for the vegetables and therefore preferred a genotype with a good taste [35]. In addition to that, a soft genotype was preferred by farmers because it takes a short time to cook.

## Conclusion

There were no differences in traits and genotypes of *S. aethiopicum*, Shum group preferred by men and women. The most considered farmer traits are seed and leaf yield, followed by pest and diseases resistance. The overall preferred genotype in terms of disease and pest resistance, leaf yield, leaf texture, and seed yield were E12 followed by E11. Whereas E12 emerged the best overall based on visual scores, its taste may not be liked by consumers. Aside from E12, the most preferred genotype was the green genotype (E11) for market and the purple genotype (E15) for personal consumption. This shows a mismatch between consumer and farmer preferences hence a need for a keener understanding of perceptions across the value chain. Thus, sensory evaluations should always accompany visual observations because genotypes excelling for physical/visual attributes may not meet the sensory preferences.

## Supplementary Information

**Additional file 1.** Initial conditional approval.

**Additional file 2.** Final approval.

**Additional file 3.** Copy of farmers consent form.

**Additional file 4.** Study tools.

## Data Availability

The data can be availed on request.
