# Supplementary material for: Farmer preferred traits and genotype choices in Solanum aethiopicum L., Shum group
Source: J Ethnobiol Ethnomed. 2021 Apr 13;17:27. doi: 10.1186/s13002-021-00455-y (PMC8042716; doi:10.1186/s13002-021-00455-y)
Supplement: Supplementary file 1 — Additional file 1. Initial conditional approval. [file 13002_2021_455_MOESM1_ESM.pdf]

UGANDA CHRISTIAN UNIVERSITY

A Centre of Excellence in the Heart of Africa

UG REC-026

1st August, 2019

To: Brenda Nakyewa

+256706339381

RE: CONDITIONAL UCUREC APPROVAL LETTTER

In reference to your Research Number UCUREC-2020-15, your Research has been given a conditional approval. You are required to submit all work through the National Research Information Management System (NRIMS) as mandated by Uganda National Council for Science and Technology (UNCST).

You are conditionally proved to proceed with data collection and obtain full approval after you have completed your submission through that process. This is to ensure your Research work is captured in the system.

A detailed approval with attachments and other specifics will be then be given to after.

Kindly work on that with urgency and obtain the approval.

Yours sincerely,

....

Prof. Peter Waiswa,

Chairpersion, UCUREC

pwasiwa@musph.ac.ug, **+256(0)772 405 357**
