# Supplementary material for: Farmer preferred traits and genotype choices in Solanum aethiopicum L., Shum group
Source: J Ethnobiol Ethnomed. 2021 Apr 13;17:27. doi: 10.1186/s13002-021-00455-y (PMC8042716; doi:10.1186/s13002-021-00455-y)
Supplement: Supplementary file 2 — Additional file 2. Final approval. [file 13002_2021_455_MOESM2_ESM.pdf]

UGANDA CHRISTIAN UNIVERSITY

A Centre of Excellence in the Heart of Africa

28/07/2020

To: Brenda Nakyewa

Uganda Christian University
0706339381

Type: Initial Review

Re: UCUREC-2020-15: Influence of Market Entry Requirements on Men and Women Participation in African Eggplant Vegetable Seed Production and Marketing, Proposal, 2020-06-30

I am pleased to inform you that the Uganda Christian University REC, through expedited review held on **07/05/2020** approved the above referenced study.
Approval of the research is for the period of **28/07/2020** to **28/07/2021.**

As Principal Investigator of the research, you are responsible for fulfilling the following requirements of approval:

1. All co-investigators must be kept informed of the status of the research.
2. Changes, amendments, and addenda to the protocol or the consent form must be submitted to the REC for re-review and approval **prior** to the activation of the changes.
3. Reports of unanticipated problems involving risks to participants or any new information which could change the risk benefit: ratio must be submitted to the REC.
4. Only approved consent forms are to be used in the enrollment of participants. All consent forms signed by participants and/or witnesses should be retained on file. The REC may conduct audits of all study records, and consent documentation may be part of such audits.
5. Continuing review application must be submitted to the REC **eight weeks** prior to the expiration date of **28/07/2021** in order to continue the study beyond the approved period. Failure to submit a continuing review application in a timely fashion may result in suspension or termination of the study.
6. The REC application number assigned to the research should be cited in any correspondence with the REC of record.
7. You are required to register the research protocol with the Uganda National Council for Science and Technology (UNCST) for final clearance to undertake the study in Uganda.

The following is the list of all documents approved in this application by Uganda Christian University REC:

UGANDA CHRISTIAN UNIVERSITY
APPROVED
2 [illegible] JUL 2020
RESEARCH ETHICS COMMITTEE

No.	Document Title	Language	Version Number	Version Date
1	Admission letter	English	4	2020-07-01
2	Informed Consent forms	English	3	2020-06-30
3	Data collection tools	English	2	2020-06-30
4	Protocol	English	Proposal	2020-06-30

Yours Sincerely

Peter Waiswa
For: Uganda Christian University REC

UGANDA CHRISTIAN UNIVERSITY
APPROVED
[illegible]
RESEARCH ETHICS
COMMITTEE
