# Supplementary material for: Farmer preferred traits and genotype choices in Solanum aethiopicum L., Shum group
Source: J Ethnobiol Ethnomed. 2021 Apr 13;17:27. doi: 10.1186/s13002-021-00455-y (PMC8042716; doi:10.1186/s13002-021-00455-y)
Supplement: Supplementary file 3 — Additional file 3. Copy of farmers consent form. [file 13002_2021_455_MOESM3_ESM.pdf]

Consent Form for Participant

[redacted]

Project Title: Influence of Market Entry Requirements on Men and Women Participation in African Eggplant Vegetable seed Production and Marketing.

I have read the information sheet for participants for this study and have had the details of the study explained for me. My questions about the study have been answered to my satisfaction, and I understand that I may ask further questions at any time.

I also understand that I am free to withdraw from the study at any time, or to decline to answer any particular Questions in the study. I agree to provide information to the researchers under the conditions of confidentially set out on the information sheet. I agree to participate in this study under the conditions set out in the information sheet from.

Signed……………………………………………………………………………...….

Name …………………………………………**Date** ………………………………………

Principle Investigator/Researcher's Name: Nakyewa Brenda, Mobile:0706339381

Co-Investigator's/Supervisor's Name: Prof. Elizabeth Kizito Balyejusa, Uganda Christian University, Department of Agricultural and Biological Sciences, Mobile: 0752141377 or Dr. Godfrey Sseremba, E-mail:gsseremba16@gmail.com
