# Supplementary material for: Farmer preferred traits and genotype choices in Solanum aethiopicum L., Shum group
Source: J Ethnobiol Ethnomed. 2021 Apr 13;17:27. doi: 10.1186/s13002-021-00455-y (PMC8042716; doi:10.1186/s13002-021-00455-y)
Supplement: Supplementary file 4 — Additional file 4. Study tools. [file 13002_2021_455_MOESM4_ESM.pdf]

Genotype Scoring guide for farmers

Trial site:…................... Planting date:….................................

**Enumeration details:**	Enumerator Name…...		Date:…..................................
**Farmer xtics:**	Farmer Name:….........................	Village….....................	Parish:…...............................
Phone number:	Age:…......	Sex:…...... (M or F)	Sub-county:…......................
…...........................	Role in value chain:…............................... (e.g., vegetable farmer, seed producer, market vendor, transporter, extensionist)		
	Position in household…................................ (e.g., head, spouse, child)		

10 genotypes selected in order of preference (Ranked: 1>2>3>4>5>6>7>8>9>10)

		**WELL WATERED**										**DROUGHT STRESSED**									
**Rep**	**Variable**	1	2	3	4	5	6	7	8	9	10	1	2	3	4	5	6	7	8	9	10
1	Disease resistance																				
1	Pest resistance																				
1	Drought tolerance																				
1	Plant Vigor																				
1	Leaf yield																				
1	Seed yield																				
1	Taste (cooked)																				
		1	2	3	4	5	6	7	8	9	10	1	2	3	4	5	6	7	8	9	10
2	Disease resistance																				
2	Pest resistance																				
2	Drought tolerance																				
2	Plant Vigor																				
2	Leaf yield																				
2	Seed yield																				
2	Taste (cooked)																				
		1	2	3	4	5	6	7	8	9	10	1	2	3	4	5	6	7	8	9	10
3	Disease resistance																				
3	Pest resistance																				
3	Drought tolerance																				
3	Plant Vigor																				
3	Leaf yield																				
3	Seed yield																				
3	Taste (cooked)																				

Notes on genotype ranking by farmers

1. A farmer's selection is judgemental

2. The farmer will select the best 10 genotypes per replication.
3. The selection is on plot basis (each plot is a genotype)
4. The farmer is guided by an enumerator to locate a replication.
5. Enumerators will be briefed on what to do and not to do to ensure that the farmer decides him/herself on the ranking of genotypes.
6. The taste (cooked) variable, leaf samples will be harvest and cooked, then allow farmers to individually taste and rank genotype preference
7. Target number of farmers: 40-50 farmers per site (including representation men, women, youth, specialists in fresh leaf produce, specialists in seed production)

Focus group discussion (FGD) guide

FGD notes

a. A focus group discussion (FGD) will be held after the individual farmer ranking of varieties (preferably after data analysis).
b. The FGD will serve as a way of giving feedback to the community on variety preferences.
c. The FGD will also serve to validate/explain the findings from individual farmer selections.
d. FGDs will be convened at each site, where men and women could be separated. In each category, a representation of different value chain actors (fresh produce or seed) will be ensured.
e. Target FGD composition: Minimum of 8 and maximum of 10 participants with representation of both fresh leaf produce and seed farmers.

FGD guide

1. Which traits would you prefer in a Nakati variety? For fresh produce (leaf)? For seed?
2. Which diseases do you experience in Nakati? How do diseases affect you as a fresh produce/seed farmers? How do you decide that a variety is disease resistant? Did you find any disease resistant variety during selections? For fresh produce? For seed?
3. Which pests do you experience in Nakati? How do pests affect you as a fresh produce/seed farmers? How do you decide that a variety is pest resistant? Did you find any pest resistant variety during selections? For fresh produce? For seed?
4. Does drought affect you as fresh produce/seed farmers? How does drought affect you as a fresh produce/seed farmer? How do you decide that a variety is drought tolerant? Did you find any drought tolerant variety during selections? For fresh produce? For seed?
5. Apart from diseases, pests and drought challenges, which other Nakati production and marketing constraints do you consider important as fresh produce/seed farmers?

Thank you for your time and volunteering of the information.
